# Uniformly Porous Nanocrystalline CaMgFe_1.33_Ti_3_O_12_ Ceramic Derived Electro-Ceramic Nanocomposite for Impedance Type Humidity Sensor

**DOI:** 10.3390/s16122029

**Published:** 2016-11-30

**Authors:** Ashis Tripathy, Sumit Pramanik, Ayan Manna, Hanie Nadia Shasmin, Zamri Radzi, Noor Azuan Abu Osman

**Affiliations:** 1 Centre for Applied Biomechanics, Department of Biomedical Engineering, University of Malaya, Kuala Lumpur 50603, Malaysia; ayanbabu@siswa.um.edu.my (A.M.); hanie_nadia@um.edu.my (H.N.S); 2 Department of Paediatric Dentistry & Orthodontics, Faculty of Dentistry, University of Malaya, Kuala Lumpur 50603, Malaysia; zamrir@um.edu.my

**Keywords:** nanoceramic, moisture, porosity, mechanism, resistive, recovery, sensitivity, long-term stability

## Abstract

Since humidity sensors have been widely used in many sectors, a suitable humidity sensing material with improved sensitivity, faster response and recovery times, better stability and low hysteresis is necessary to be developed. Here, we fabricate a uniformly porous humidity sensor using Ca, Ti substituted Mg ferrites with chemical formula of CaMgFe_1.33_Ti_3_O_12_ as humidity sensing materials by solid-sate step-sintering technique. This synthesis technique is useful to control the grain size with increased porosity to enhance the hydrophilic characteristics of the CaMgFe_1.33_Ti_3_O_12_ nanoceramic based sintered electro-ceramic nanocomposites. The highest porosity, lowest density and excellent surface-hydrophilicity properties were obtained at 1050 °C sintered ceramic. The performance of this impedance type humidity sensor was evaluated by electrical characterizations using alternating current (AC) in the 33%–95% relative humidity (RH) range at 25 °C. Compared with existing conventional resistive humidity sensors, the present sintered electro-ceramic nanocomposite based humidity sensor showed faster response time (20 s) and recovery time (40 s). This newly developed sensor showed extremely high sensitivity (%S) and small hysteresis of <3.4%. Long-term stability of the sensor had been determined by testing for 30 consecutive days. Therefore, the high performance sensing behavior of the present electro-ceramic nanocomposites would be suitable for a potential use in advanced humidity sensors.

## 1. Introduction

A variety of techniques have been subsisted for detection of relative humidity (RH) in various systems or devices. RH is one of the most frequently influenced physical parameters in every field of life, e.g., domestic, pharmaceuticals, electronics, textile, biomedical field, and laboratories [1,2,3,4,5,6,7]. In biomedical field, sweating is one of the great discomforts to the patients, who have to use stumps or prosthetic devices for longer period. However, estimation of sweating rate is a great challenge to the researchers at the narrow zone between the socket and skin [8]. In response to the high demands of RH in wide field of applications, it is now essential to develop proper RH sensors with lower cost; faster, simpler, and more unswerving detection; and higher sensitivity. Nanostructured materials significantly promote the humidity sensing properties in contrast to the conventional porous ceramic materials owing to ultra-high surface-to-volume ratio, small grain size and very large boundary areas [1,2,3,4,9]. Some ceramic materials using precision capacitive technique also showed rapid dynamic responses as well as high temperature compensation [10,11]. Thus, the nanomaterials display better sensing behavior, such as smaller response time, shorter hysteresis, improved sensitivity and better stability [9]. Therefore, various different nanomaterials have been explored as potential sensing elements for humidity-measuring systems [9,12,13,14]. In this context, ceramic oxides showed several beneficial properties, such as thermal, physical and chemical stabilities, mechanical strength, and so on [15]. For humidity sensing, ceramics are used mainly as porous sintered form [16]. Ceramic sensors mainly depend on their surface characteristics [17]. Therefore, maintaining the porous structure with high surface activity is a great concern to researchers to develop ceramic sensors with suitable electrical characteristics related to humidity sensors [18,19].

In this context, lead-free ferroelectric ceramics (e.g., BiFeO_3_) can show spontaneous high polarization and high magnetic Curie temperature [20]. Further, crystalline tin oxide (SnO_2_) based flexible composite had shown good resistive sensitivity for humidity and gas sensor [21]. Recently, more interesting studies on humidity sensors of different spinel-type ceramics were investigated. Magnesium (Mg) based spinel ferrite has been shown to be one of the best potential spinel ceramic candidates for resistive type relative humidity sensors [22]. Major advantages of these materials are long-term stability, high porosity, and that they can cover wide relative humidity range. Thus, these advantages make the resistive sensors suitable to use for controlling and displaying the products in industrial, commercial, and residential applications [8]. However, sometimes, few problems associated with oxide materials, including long-term drift, slow down the response time and adsorption/desorption hysteresis. In this context, Mg ferrite is oxygen deficient material at lower sintering temperature as well as porous, which is desirable for humidity sensing [23]. Since defective sites on the surface are highly reactive due to unsaturated bonds, it helps water vapors to dissociate [24,25]. Recent studies on this material investigate improving the sensitivity and minimizing the response time by varying the size of the synthesized nanoparticles [26,27].

Substitution of cations in magnetic and non-magnetic ferrites has ability to change their magnetic or electrical properties [28,29,30]. The change in physical and chemical properties may be found in the ion substituted spinel ferrites due to the distribution of the cations in their available trihedral (named as A-site) and octahedral (named as B-site) sites [31,32,33]. In order to improve the electrical and magnetic properties of magnesium ferrites, various cations had been investigated [34]. In different studies, researchers showed that the incorporation of Ti ion could reduce the electrical conductivity of the Mg–Zn ferrite [35], Li-ferrite [36] and Co–Zn ferrite [37]. Moreover, inclusion of Ti^4+^ ions reduced the dielectric constants in Ni–Zn ferrite [38] and influenced the magnetic properties of Mg–Zn [39] ferrites. The substitution of Ti^4+^ ion at iron sites in manganese (Mn) ferrites was found to be effective in order to minimize the magneto crystalline anisotropy and to enhance the electrical resistivity. The reduction in conductivity and dielectric loss of ferrites is very important for humidity sensor applications [40].

The above survey indicates that the use of Ca, Ti doped magnesium ferrite nanocomposite might be potential candidate as an effective humidity sensor material. To the best of our knowledge, there is no existing report by any other group on the synthesis of Ca, Mg, and Ti oxide based complex ferrite materials. Therefore, for the first time, we aim to synthesize Ca, Mg, and Ti oxide based complex ferrite material (probable empirical formula CaMgFe_1.33_Ti_3_O_12_) using inexpensive solid-state step-sintering from inexpensive iron oxide (Fe_2_O_3_), magnesium carbonate (MgCO_3_), calcium oxide (CaO) and titania (TiO_2_) powders and by optimizing the sintering conditions for humidity sensing application in the field of biomedical engineering. Since our previous report on capacitive sensor have shown successful effort [41], it has motivated us to carry out humidity dependent electrical impedance, complex impedance, modulus, sensitivity, linearity, hysteresis, and response and recovery time analysis measurements on nanostructured CaMgFe_1.33_Ti_3_O_12_ complex ceramic samples in the present study.

## 2. Experimental Details

### 2.1. Preparation of Sensing Material

The CaMgFe_1.33_Ti_3_O_12_ nanocomposite was developed by solid-state step-sintering at suitable temperatures in a box furnace (XY1600, Nanyang Xinyu Furnaces, He’nan, China) using commercial ceramic powders of CaO, MgCO_3_, Fe_2_O_3_ and TiO_2_ (99.9% pure, Fisher scientific Ltd., Selangor, Malaysia) with proper molar ratio, as reported before [41]. The empirical formula of the unsintered nanocomposite was calculated to CaMgFe_1.33_Ti_3_O_12_ from the used molar ratio of the raw ingredients. All the ingredients powders were first, dry mixed thoroughly in normal grinder for 1 h and then, the mixed powders were ball-milled in aqueous medium of 70% alcohol in a ball mill (PM200, Retsch, Düsseldorf, Germany) at 300 rpm for 72 h. The mixed slurry was kept at 250 °C for 5 h for self-combustion followed by drying at 220 °C in a programmable oven for 48 h. The cylindrical pellets (Φ10 mm × 1–2 mm) were prepared at a maximum uniaxial pressure of 450 MPa under a hydraulic press (GS15011, Graseby Specac, Kent, UK). The testing pellets were step-sintered in ambient gas atmosphere. Since, according to our previous studies, morphological, physical, and dielectric properties suggested that the material of sintering condition 1050 °C was best, only pellets sintered at 1050 °C were used as resistive or impedance type humidity sensors in the present investigation. In this particular sintering condition, mainly three steps were involved. First, the compact pellets were heated at 350 °C for 1 h at a ramp rate of 5 °C/min, then they were calcined at 550 °C for 3.5 h at a ramp of 10 °C/min, and finally, the pellets were sintered at 1050 °C for 1.3 h at a rate of 15 °C/min. After the final step, it was cooled down to 750 °C at 20 °C/min with a holding time of 3 h and then allowed to normal furnace cooling up to the room temperature (i.e., 25 °C). The solid-state step-sintering ensured the desired ranges of particle size, pore size, and porosity.

### 2.2. Humidity Sensor Fabrication and Measuring Setup

**Figure 1:**
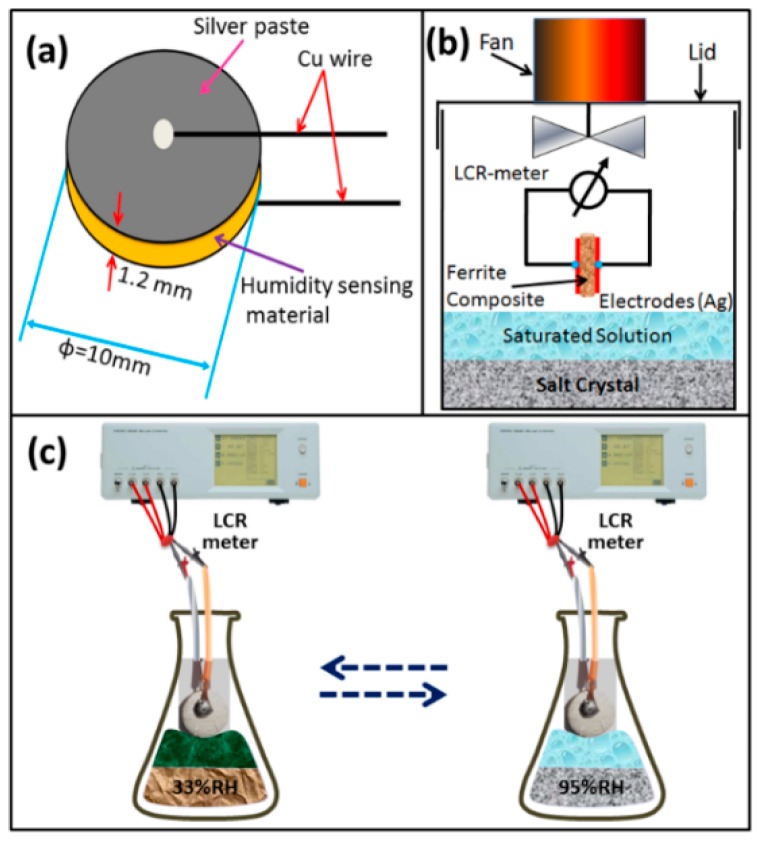
Schematics of: (**a**) ceramic pellet with silver electrodes; (**b**) experimental setup for relative humidity (RH) sensing measurement; and (**c**) humidity sensitive capacitive device at different RHs.

A measured amount of the dried milled powders of CaMgFe_1.33_Ti_3_O_12_ was uniaxially pressed at high pressure of 450 MPa for 4 min to form the pellets of 10 mm in diameter and about 1.2 mm in thickness (Figure 1a). Then, the pellets were sintered at the condition of 1050 °C, as mentioned above. The silver electrodes were screen printed on both sides of each sintered pellet and two copper (Cu) wires were connected to both side-silver electrodes (see Figure 1a). Finally, the fabricated sensor was placed for aging at 150 °C in air for 1 h followed by stabilization at 1 V voltage in 95% RH for 24 h. The sensor element (silver coated sintered electro-ceramic nanocomposite disc) was then placed in a thermostatic vessel and exposed to different RHs (see Figure 1b). The RHs between 33% RH and 95% RH (see Figure 1c) were obtained as the humidity generation source using saturated salt solutions such as MgCl_2_, Mg(NO_3_)_2_, NaCl, KCl and KNO_3_, which would produce the environments with different RHs of 33%, 55%, 75%, 85% and 95% RH, respectively at 25 °C.

X-ray diffraction (XRD) results were obtained by using X-ray diffractometer (Empyrean, PANalytical, Almelo, The Netherlands). The microstructure of the samples was analyzed by field emission-scanning electron microscopy (FESEM, AURIGA, Carl Zeiss, Jena, Germany). Pore size distribution (PSD) was computed by the ImageJ from the corresponding FESEM images. Density (ρ in g/cc), open porosity (%), and absorbed water or water absorption (%) of the porous materials were evaluated by modified Archimedes’ principle, as explored in our previous studies [42,43,44] using distilled water at 25 °C.

The impedance response to RH of CaMgFe_1.33_Ti_3_O_12_ nanoceramic derived sintered electro-ceramic nanocomposite humidity sensor was studied by impedance spectroscopy (IS) (3532-50 LCR Hi tester, Hioki) using alternating current (AC) frequency, ranging from 100 Hz to 1 MHz at 25 °C. The sintered electro-ceramic nanocomposite used as sensing element and the schematic of humidity sensing measuring equipment are depicted in Figure 1a,b, respectively. After each change of the humidity, the sensor element was exposed to the new humidity for 2 min, before measuring of the new resistance. The humidity response and recovery times were carried out over the resistance changes when the RH was changed during humidification (33% to 95% RH) as well as during desiccation (95% to 33% RH) at 25 °C. To determine stability of the sensors, the resistivity (due to RH ranging 33% to 95% RH) vs. time characteristics were measured at 25 °C, for 30 days in in 2-day intervals.

## 3. Results and Discussion

### 3.1. Structural and Morphological Analysis

**Figure 2:**
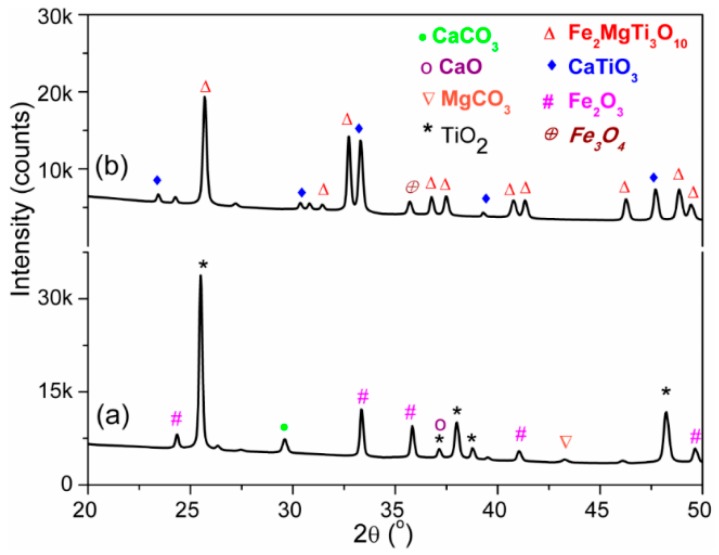
X-ray diffraction (XRD) patterns of: (**a**) unsintered; and (**b**) sintered (at 1050 °C) CaMgFe_1.33_Ti_3_O_12_ nanoceramic composites. X-ray source was Cu-Kα radiation, and 2θ was 20°–50°. Note: the different colored planes represent the crystalline planes of respective materials: pink, Fe_2_O_3_; black, TiO_2_; dark red, MgCO_3_; green, CaCO_3_; purple, CaO; red, Fe_2_MgTi_3_O_10_; blue, CaTiO_3_; and brown, Fe_3_O_4_.

X-ray diffraction (XRD) patterns of unsintered and sintered (at 1050 °C) ceramic materials are depicted in Figure 2a,b. Presence of almost all the used raw materials was observed in XRD-pattern of unsintered ceramic mixture. The major crystalline peaks shown by anatase TiO_2_ (Powder diffraction file (PDF) No. 98-015-4609) and ferrite Fe_2_O_3_ (PDF No. 01-084-0308) are indicated in Figure 2a. Other small peaks of CaO and MgCO_3_ have also been detected. A new peak of calcium carbonate (CaCO_3_, PDF No. 01-072-1650) at 2θ = 29.59° indicates that the CaO and MgCO_3_ had reacted during mechano-chemical mixing. After sintering, the unsintered CaMgFe_1.33_Ti_3_O_12_ nanoceramic converted into mainly two new phases, which were resembled with the standard XRD patterns of orthorhombic armalcolite (Fe_2_MgTi_3_O_10_, PDF No. 00-013-0353) and perovskite CaTiO_3_ (PDF No. 00-008-0092) at 1050 °C, are depicted in Figure 2b. The crystallite size (t) of the sintered material was estimated by modified Debye Scherer formula in Equation (1) [42]:

(1) $$t=\frac{\kappa \lambda }{\Delta \theta_{\mathrm{FWHM}}\cdot \cos\theta -4\epsilon \cdot \sin\theta }$$

where Δθ_FWHM_ is the full width at half maxima of the XRD peak (in radian), κ is a constant (~0.9) that depends on the particle morphology, λ is the x-ray wavelength (λ_CuKα_ = 1.540546Å), ϵ is the crystal strain or elastic residual strain, and θ is the Braggs’ diffraction angle (in degree).

**Figure 3:**
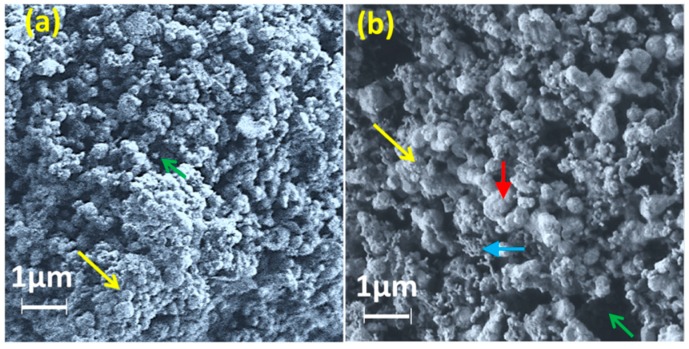
Scanning electron micrographs of the specimens for: (**a**) unsintered; and (**b**) sintered at 1050 °C. Note: the green and yellow arrows indicate pores and particles; and vertical red and horizontal blue arrows indicate the armalcolite (average size: 685 nm) and perovskite (<100 nm) structure phases, respectively. The grain and grain boundary are also clearly revealed at the large size armalcolite phase.

The two different crystallite sizes of the sintered materials at 2θ =25.70° for (101) plane of Fe_2_MgTi_3_O_10_ and 2θ = 33.32° for (440) plane of CaTiO_3_ are 20.7 nm and 5.9 nm, respectively. This result clearly indicates that the material sintered at 1050 °C contained mainly two phases, which were also observed in the scanning electron micrograph (see Figure 3). Interestingly, at 2θ = 35.72°, another new peak of Fe_3_O_4_ (PDF No. 01-088-0315) for (311) plane clearly indicates the transformation of Fe^3+^ into Fe^2+^ from Fe_2_O_3_ after sintering at 1050 °C. According to the peak areas of the corresponding peaks, the amounts of Fe_2_MgTi_3_O_10_, CaTiO_3_ and Fe_3_O_4_ present in the sintered material are 85.80%, 12.16% and 2.04%, respectively (see Table 1, with respect the total area of peak).

Lattice parameters of the orthorhombic armalcolite (Fe_2_MgTi_3_O_10_) and cubic perovskite (CaTiO_3_) can be calculated using Equations (2) and (3), respectively, and the values are illustrated to Table 2. Since only one unique peak was shown by Fe_3_O_4_ at 2θ = 35.72°, the lattice parameters were not calculated in the present study:

(2) $${\left[\frac{1}{d_{\mathrm{hkl}}^{2}}\right]}_{\mathrm{orthorhombic}}=\frac{h^{2}}{a^{2}}+\frac{k^{2}}{b^{2}}+\frac{l^{2}}{c^{2}}$$

(3) $${\left[\frac{1}{d_{\mathrm{hkl}}^{2}}\right]}_{\mathrm{cubic}}=\frac{h^{2}+k^{2}+l^{2}}{a^{2}}$$

where d_hkl_ is the inter-planer distance between the planes (hkl); and *a*, *b* and *c* are unit cell parameters. It was found that unit cell volume of both the crystal lower than that of standard unit cells of the corresponding crystals. It was affected by lattice strain. The interesting result obtained by the XRD was that the unit cell volume of the CaTiO_3_ was higher than that of Fe_2_MgTi_3_O_10_, which was reverse of the particle size obtained from the SEM micrograph of the sintered material (see Figure 3). This result indicates that the particles of CaTiO_3_ obtained at the 1050 °C sintering condition were not grown completely. This is an extremely advantageous technique to control the second phase materials according to desired properties.

A submicro-porous structure of the ceramic nanomaterials is depicted in the scanning electron micrographs of Figure 3. Average particle size of the unsitered ceramic (210 nm, see Figure 3a) increased after sintering at 1050 °C (630 nm, see Figure 3b). The average pore size in the specimen after sintering at 1050 °C was found as 850 nm. Two different sizes (685 nm and < 100 nm) in the sintered materials distinctly indicate the development of two distinct phases such as Fe_2_MgTi_3_O_10_ and CaTiO_3_ (see Figure 3b). The smaller size (typically <100 nm) particles were the perovskite CaTiO_3_ phase and the large particles were Fe_2_MgTi_3_O_10_ phase particles (average size of 685 nm). They were also confirmed by XRD study. The grain boundaries and grains were clearly revealed in the sintered materials at higher magnifications, as found at the large size armalcolite phase in Figure 3b.

**Figure 4:**
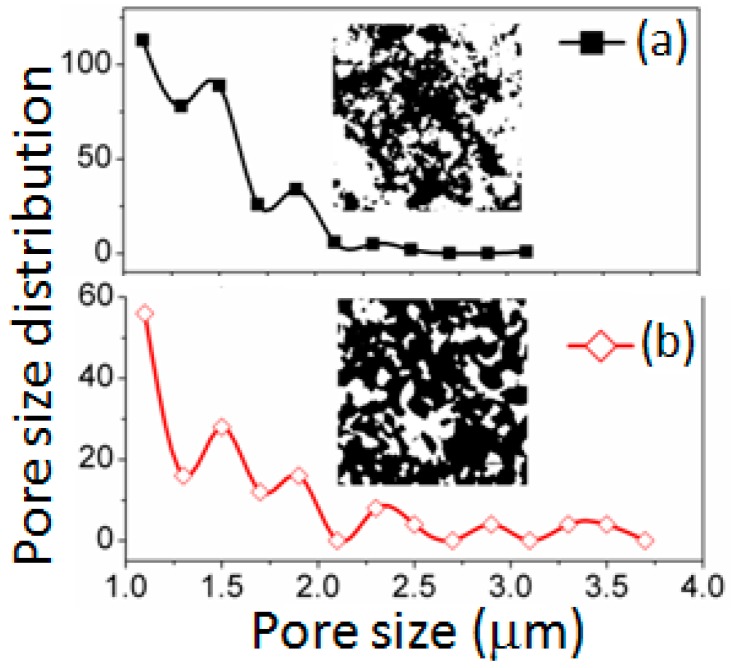
Pore size distribution (PSD) plots of: (**a**) unsintered; and (**b**) sintered (at 1050 °C) materials quantifying from the respective Inverted SEM figures using ImageJ. The corresponding Inverted SEM image is also presented as Inset image for both the materials. The black analyzed areas in the Insets indicate pores and white areas represent the particles.

Figure 4 shows the PSD of unsintered and sintered (at 1050 °C) materials estimated from the Inverted SEM figures employing ImageJ. The unsintered material (see Figure 4a) showed almost bimodal or single modal pore distribution with a PSD less than 1.5 μm. However, at sintering condition (at 1050 °C, see Figure 4b), the maximum pore size was also found to be more than 3.5 μm. This result suggests that larger size open pores had been developed in sintered materials. The multimodal PSD at 1050 °C implies that the three different types of cluster developed by three distinct structural phases, including armalcolite, perovskite, and ferrite, which were confirmed by XRD study (see Figure 2), were inter connected. Hence, the multimodal PSD present in the sintered material with higher pore size would be responsible to possess a higher resistive sensitivity due to RH change. Large sized pores (above 850 nm) distributed along the agglomerated grains were observed in the sintered sample. These pores played vital role for faster response of sensor since the rate of water adsorption was controlled by the rate of diffusion of water vapors. These typical porous structures present in our sintered electro-ceramics could readily display the adsorption and condensation of water vapors from the sensor devices. Moreover, this structure is an excellent beneficial for the moisture absorption. The unsintered sample showed insufficient porosity with higher density, and thus, could not use for further study. In the oxide based porous compounds, including the Mg-ferrite in our case, the water vapor molecules would react with the grains of material’s surface as well as with the grains present inside the material of sensing element. In addition, a larger specific surface area (sintered at 1050 °C) indicated a larger active surface exposed to the gas and a higher sensitivity to water vapor or gaseous molecules. The specific surface area (15–25 m^2^/g) of the unsintered sample could partially be the effect of large amount of closed pores in order to get detriment in open pores. The obtained changes in the both particle size and specific surface area suggest that the used step sintering technique, as a result of the formation of the different secondary or tertiary phases, inhibited the crystal growth and favored the increase in specific surface area. On the other hand, the sintered electro-ceramic showed the higher porosity (>40%), lowest density, the largest specific area (10–70 m^2^/g) and a wide PSD in comparison of unsintered CaMgFe_1.33_Ti_3_O_12_ nanoceramic. The large specific area enhanced active surface to adsorb or desorb water vapors and, thus, improved the resistive sensitivity with the variation of RH. Further, since large pores are necessary for a rapid response, this sintered electro-ceramic can easily improve the adsorption and condensation of water vapor [45]. Hence, the sintered electro-ceramic (at 1050 °C) was considered as the ideal condition for our further humidity sensing analysis.

The bulk density was conducted in order to measure the open porosity of the materials. The unsintered and sintered (1050 °C) materials showed bulk density of 1.989 ± 0.091 g/cc and 0.941 ± 0.035 g/cc, respectively; and the open porosity of unsintered and sintered (1050 °C) materials was 73.84% ± 1.24% and 40.26% ± 1.33%, respectively. The decreased value of density showed that the densification was significantly controlled by sintering condition. Densification was controlled using step-sintering conditions to enhance the porosity. The bulk density of the nanocomposite sintered at 1050 °C was significantly (50%) decreased due to lattice diffusion phenomenon during sintering with keeping substantial porosity [46]. The porous morphology (see Figure 2b) and PSD (see Figure 3b) of the sintered (at 1050 °C) nanocomposite also strongly supported this result. The water absorption indicated the total porosity present in the newly developed materials. The uniform high porosity of the sintered sample was obtained owing to the novel preparation technique (by solid-state reactions during step-sintering, rather conventional chemical routes). The high water absorption (~67%) in the sintered sample suggested the uniform porosity that was also revealed in the SEM-image (see Figure 2b). Therefore, from the above structural and physical studies, the sintered nanocomposite (at 1050 °C) had been considered as a potential humidity sensing material in our further study [8,47,48,49].

### 3.2. Humidity Sensing Measurements

**Figure 5:**
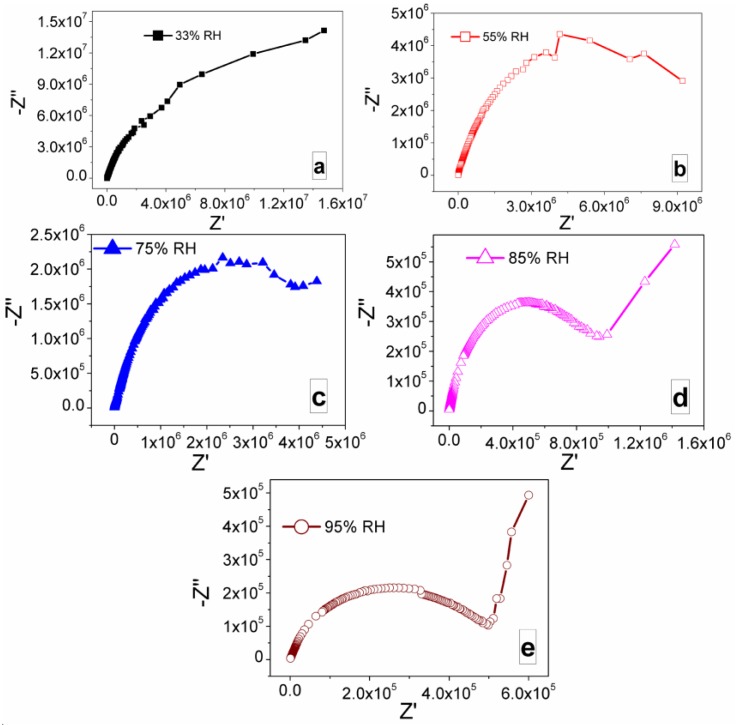
Nyquist plots of sintered electro-ceramic nanocomposite device at: (**a**) 33% RH; (**b**) 55% RH; (**c**) 75% RH; (**d**) 85% RH; and (**e**) 97% RH. Note: At lower RH (33%, 55% and 75% RH) (**a**–**c**) the semicircles are formed and the curvature of the semicircle decreases with increasing RH, as a result the value of intrinsic impedance decreases, which is mainly due to the interaction between the sensing material and water particles. With elevating of RH from 85% to 95%, a linear curve appeared in the low-frequency range and the semicircle became smaller (**d**,**e**). The ionic and/or electrolytic conductivity played crucial role in the formation of straight line in case of complex impedance plot.

The complex impedance spectra of the newly developed CaMgFe_1.33_Ti_3_O_12_ nanoceramic derived sintered electro-ceramic nanocomposite material based humidity sensor with different humidity conditions, i.e., 33%–95% RH, are depicted in Figure 5a–e.

It was found that the impedance value decreased with increasing of RH. This study describes the mechanism of impedance response with the function of humidity. From the SEM analysis it was confirmed that at 1050 °C sintering temperature, the porosity of the sintered nanocomposite was achieved to the desired level, which enhances the hydrophilic surface characteristics of the developed sensors material. When the porous sintered nanocomposite was placed in a humidity environment, the oxygen and water from surrounding atmosphere could be chemisorbed onto its hydrophilic surface, which played a vital role to alter the sensing characteristics of the sensor. In addition, due to increase of RH from 33% to 95%, more amount of water particles were absorbed on the composite’s surfaces. It mainly resulted to a decrease in impedance such a manner that the dielectric properties could be tailored. In Figure 5, it was observed that at lower humidity levels, one semicircle was formed and as humidity increases, a line started to appear. This line associated to electrode/interface effect. As humidity increased, the semicircle started to shrink and the line became longer. Therefore, it indicates that at higher RH value, the dielectric properties seem to be increased.

**Figure 6:**
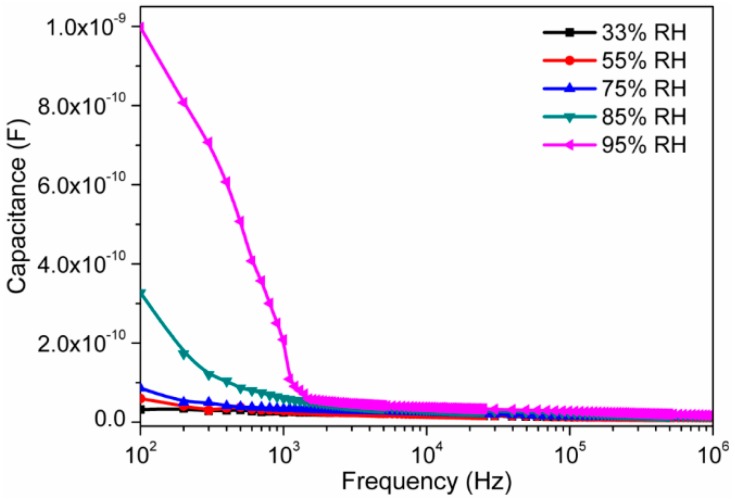
Variation of capacitance of electro-ceramic nanocomposite at different relative humidity as a function of frequency in logarithmic scale at 25 °C. Note the increase in capacitance value with increasing of RH and at higher humidity range (>85% RH), a sharp decreased response of capacitance with frequency.

To analyze the effect of humidity on our present electro-ceramic nanocomposite device, we measured the impedance, capacitance and modulus at different humidity conditions in a frequency range of 10^2^ Hz–1 MHz at 25 °C. The RH dependent capacitance effect is depicted in Figure 6. It was observed that for all the relative humidity levels, the value of capacitance decreased with increase in frequency. This decreasing rate was faster at low frequencies but slower at higher frequencies. The capacitance value was also found to be increased with increasing of humidity. As RH increased, more H^+^ ions would be available in adsorbed water layers on the surface of sensor material, and, as a result, the capacitance value becomes higher [50]. Impedance also highly depended on the value of frequency since it was directly related to energy of carriers. At lower humidity condition, due to discontinuity of water particles, much energy was required to transfer the protons from one hydroxyl group to its adjacent group via hopping mechanism. Thus, the sensors material of sintered electro-ceramic nanocomposite exhibited very high electrical impedance. However, at higher humidity condition, due to continuous water layer, the proton required very less energy to transfer the protons from one hydroxyl group to its adjacent group via hopping. This resulted to increase ionic conductivity and further decrease the electrical impedance.

**Figure 7:**
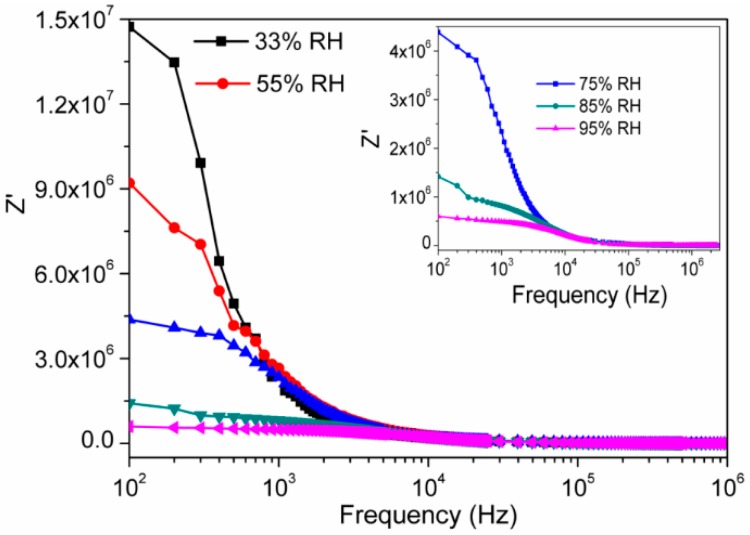
Variation of real impedance (Z′) of electro-ceramic nanocomposite at different relative humidity as a function of frequency in logarithmic scale at 25 °C. Note: As humidity increases, the value of impedance decreases and the decreased rate is fast in the lower frequency range and it becomes slower at higher frequencies (>10^4^ Hz) (inset: Magnified Z′ vs. log(*f*) response at 75%, 85% and 97% RH).

Figure 7 depicts the frequency dependent impedance (Z′) responses of the electro-ceramic nanocomposite at different RHs. It showed the decrease in impedance value with increasing frequency. The rate of decreasing in impedance was faster at lower frequency range but slow and almost constant at higher frequencies. The impedance value decreased when RH was increased. It indicates that the number of H^+^ ions is increased due to the increasing of concentration of adsorbed water, which play a vital role in conduction mechanism and gradually the mobility of ions increase.

**Figure 8:**
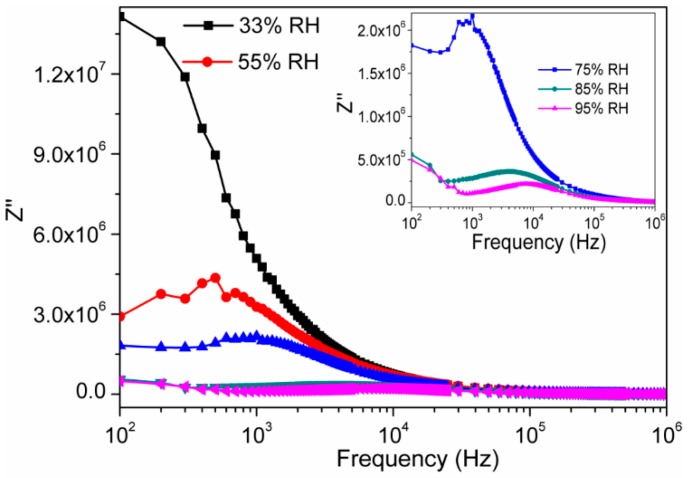
Variation of imaginary (Z″) impedance of electro-ceramic nanocomposite at different relative humidity as a function of frequency in logarithmic scale at 25 °C. Inset image represents a magnified scale of Z″ vs. log(*f*) plot of developed ceramic at higher RH (at 75%, 85% and 95% RH) with prominent relaxation peak. Note: Lower frequency relaxation peaks are observed and these peaks are more prominent at higher RH (>75% RH).

The frequency dependent capacitive reactance of the sintered electro-ceramic nanocomposite, i.e., imaginary part of the impedance (Z″), for different RHs is depicted in Figure 8. There was a peak obtained in each curve. This peak frequency was also found to shift toward right, as increasing humidity. This particular phenomenon is typically associated with the capacitance values of the materials [51].

**Figure 9:**
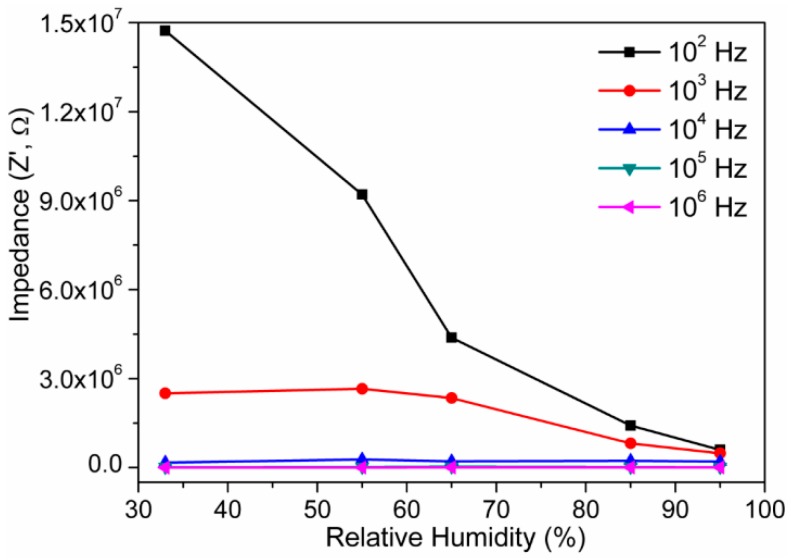
Impedance versus RH measured of electro-ceramic nanocomposite at various frequencies at 25 °C. Note: the impedance varies from 1.47 × 10^7^ Ω to 6 × 10^5^ Ω; since highest sensitivity of 0.23 MΩ/Δ% RH was found at 10^2^ Hz frequency, 10^2^ Hz was considered as most suitable test frequency in our further analyses.

Humidity dependent impedance responses at 10^2^, 10^3^, 10^4^, 10^5^, and 10^6^ Hz for sintered electro-ceramic nanocomposite sensors are depicted in Figure 9. The impedance of the electro-ceramic nanocomposite humidity device decreased with the increase in RH. At 10^2^ Hz, impedance of the nanocomposite humidity sensor device was 1.47 × 10^7^ Ω at 33% RH and it decreased to 6 × 10^5^ Ω at 95% RH. The impedance of this sensor decreased from 2.51 × 10^6^ Ω at 33% RH to 4.8 × 10^5^ Ω at 95% RH at 10^3^ Hz. At a frequency of 10^4^ Hz, the impedance changed from 2.02 × 10^5^ Ω to 1.6 × 10^5^ Ω. At a frequency of 10^5^ Hz, impedance changed from 1.7 × 10^4^ Ω to 1.6 × 10^4^ Ω. At a frequency of 10^6^ Hz, impedance changed from 2.2 × 10^3^ Ω to 5.43 × 10^2^ Ω. Therefore, it indicates that the humidity change affects the impedance considerably at intermediate and low frequencies, but the change is very small at higher frequencies. In order to evaluate the impedance characteristics of a nanocomposite sensor as a function of humidity, device sensitivity (S_Z_) can be estimated using Equation (4) [52,53]:

(4) $$S_{Z}=\frac{\Delta Z}{\Delta \%\mathrm{RH}}$$

where ΔZ is change in impedance (i.e., Z′) at the corresponding change RH, i.e., Δ% RH. From Equation (4), the sensitivity of device is 0.23 MΩ/Δ% RH (33%–95% RH), 0.032 MΩ/Δ% RH, 0.68 kΩ/Δ% RH and 0.027 kΩ/Δ% RH at 10^2^, 10^3^, 10^4^ and 10^6^ Hz, respectively. This result confirms that the highest sensitivity is observed at 10^2^ Hz. The present device sensitivity was significantly higher compared to the other existing resistive type humidity sensors (S_z_ of BaTiO_3_ thin film ~0.16 MΩ/Δ% RH, TiO_2_/Li_2_O/V_2_O_5_ based composite ~0.011 MΩ/Δ% RH, Porous ZnAl_2_O_4_ spinel nanorods ~0.08 MΩ/Δ% RH, LiZnVO_4_-doped SnO_2_ ~0.0012 MΩ/Δ% RH, ZnO nanorods ~0.082 MΩ/Δ% RH and so on, at 100 Hz) [51,54,55,56,57]. Therefore, 10^2^ Hz frequency was adopted as the testing frequency in all the succeeding analyses of the present investigation.

**Figure 10:**
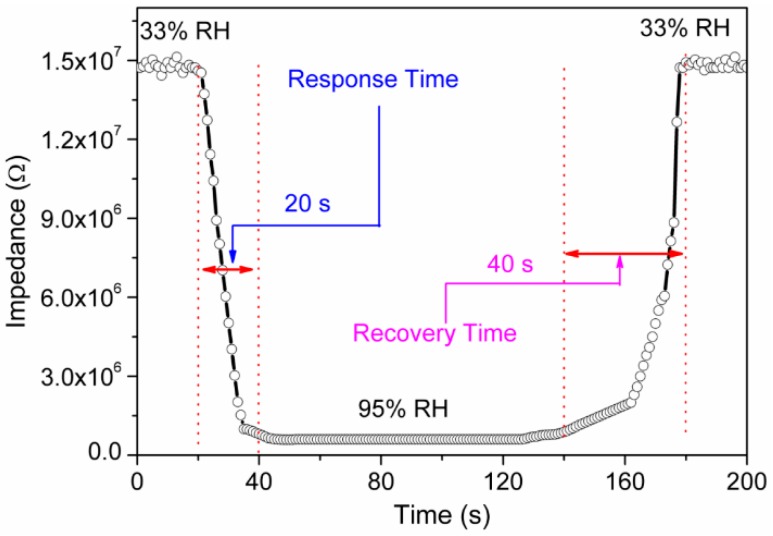
Response and recovery curve of electro-ceramic nanocomposite measured at 10^2^ Hz for humidity levels between 33% RH and 95% RH at 10^2^ Hz. Note: Response time is ~20 s and Recovery time is ~40 s.

In order to evaluate the performance of a humidity sensor, response and recovery behaviors are widely checked. The response time was measured by the time elapsed to reach 90% of the final signal at a given RH. On the other hand, the recovery time was measured by the time elapsed to come back within 10% of initial signal value. Here, response and recovery times of a sensor were measured by alternately exposing to 33% RH–95% RH. Both these times are normally very sensitive to structure and must be shortest for a practical application. In order to determine the response and recovery times, a graph of impedance versus time was plotted at AC voltage of 1 V at test frequency of 10^2^ Hz. In case of the present electro-ceramic nanocomposite sensing device, response time was 20 s when humidity was changed from 33% RH to 95% RH and recovery time was 40 s when humidity was changed from 95% RH to 33% RH, as depicted in Figure 10. Our newly developed resistive humidity sensor showed faster response and recovery times than the reported conventional sensors (e.g., response and recovery time of LiZnVO_4_-doped SnO_2_ ~60 s and ~100 s, Ba_0.5_Ni_0.5_SnO_3_ ~3 min and ~4 min, Li-doped mesoporous silica A-SBA-15 ~60 s and ~180 s, LiCl-doped mesoporous silica MCM-41 ~100 s and ~150 s, mesoporous ZnO–SiO_2_ composite ~50 s and ~100 s respectively, and so on) [51,58,59,60,61].

**Figure 11:**
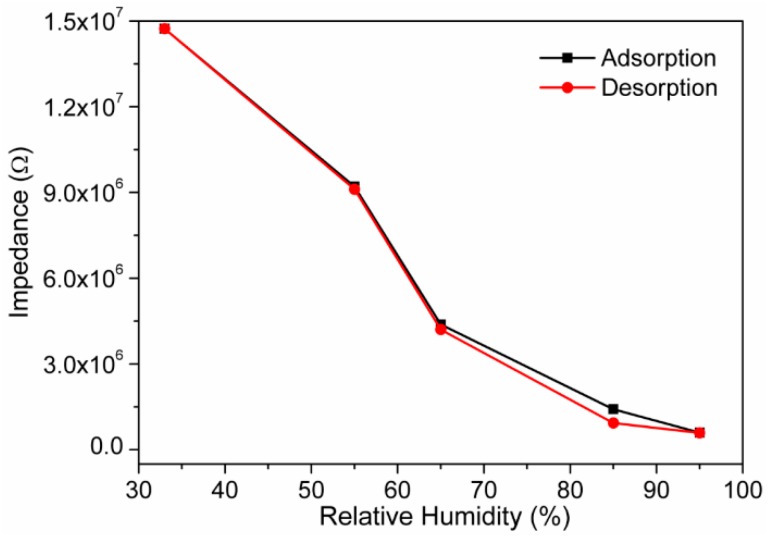
Humidity response of the sensor based on sintered electro-ceramic nanocomposite during humidification and desiccation process at 10^2^ Hz. Note: The hysteresis loss value is extremely low (~3.4%) owing to the faster rate of adsorption and desorption of water particles on the electro-ceramic surface. This hysteresis was significantly lower than other conventional sensors.

The humidity hysteresis response, which is maximum difference between the humidification and desiccation curve, is another crucial characteristic of humidity sensor. The humidity hysteresis of the present electro-ceramic nanocomposite sensor is shown in Figure 11. The maximum hysteresis response of ~3.4% at 100 Hz observed in the present electro-ceramic devices ensures the good reliability of our sensor. This hysteresis value of the present newly developed humidity sensor is significantly lower than many conventional resistive humidity sensors (LiZnVO_4_-doped SnO_2_ ~6%, CdTiO_3_ ~7%, BaTiO_3_ nanofibers ~5%, BaTiO_3_/polystyrene sulfonic sodium ~8%, and so on) [51,53,62,63].

**Figure 12:**
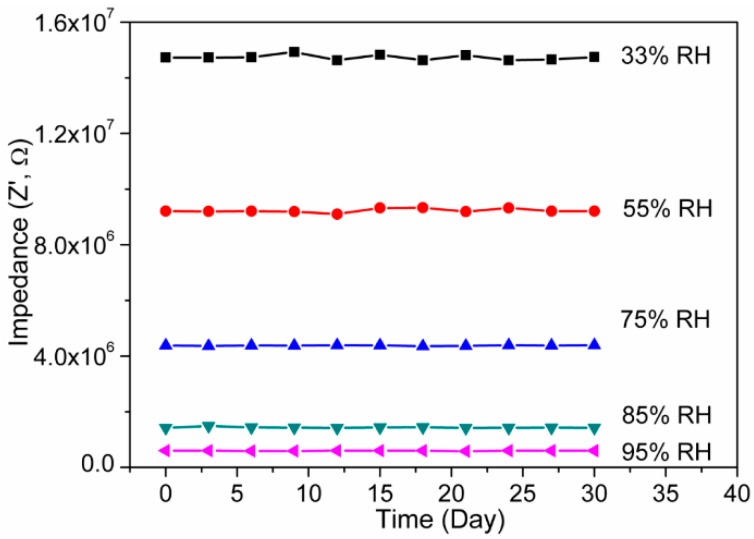
Long term stability property of the sensor based on sintered electro-ceramic nanocomposite over 30 days of measurements at 10^2^ Hz and 1 V. Note: Stability of the sensors was tested via by examining the humidity-resistivity properties at 25 °C, in the humidity range of 33%–95% RH over 30 days in a regular interval of each two days; here, no significant change was found.

In addition to the aforementioned electrical parameters, stability is another one of the most important measuring properties for determining the advantages of a humidity sensor. In order to obtain the long-term stability, the present electro-ceramic nanocomposite based humidity sensor was studied in air at 25 °C for 30 days, while impedances were measured repeatedly at two-day intervals at different RH conditions, as depicted in Figure 12. The measurements were done at 10^2^ Hz with 1 V AC power supply at 25 °C. Very little fluctuation or almost no variation in impedance of the present device was found over the 30 days of measurements. As a matter of fact, the impedance or resistance of the sensor slightly increased in ambient atmosphere due to aging operation. It evidently confirms that the present resistive sensor has significantly good stability. The increased response of conductivity with RH of the present electro-ceramic nanocomposite could be explicated by the Grotthuss mechanism [64], which can be corroborate this property by transferring of proton (H^+^) in between the water molecules through tunneling [1].

**Figure 13:**
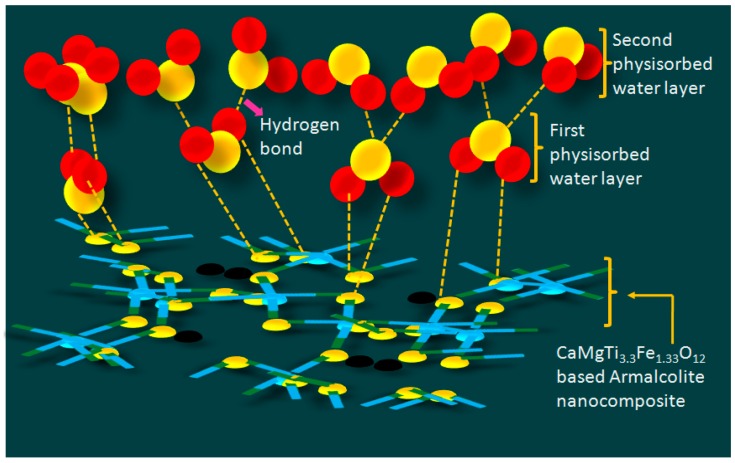
A **s**chematic humidity sensing mechanism of the sensor based on CaMgFe_1.33_Ti_3_O_12_ nanoceramic derived sintered electro-ceramic armalcolite nanocomposite at low and high humidity. Note: The water molecular adsorption on sintered electro-ceramic nanocomposite: in 1st layer, the water molecules were attached to the electro-ceramic via two hydrogen bonds, whereas, in the 2nd layer, they were adsorbed only via one hydrogen bond.

At lower RH condition, the water molecules were chemically adsorbed (chemisorption) onto the available active sites of the surface of oxide based CaMgFe_1.33_Ti_3_O_12_ nanoceramic derived sintered electro-ceramic nanocomposite via double hydrogen bonding and it is depicted in Figure 13. Due to double hydrogen bonding, the water molecules were unable to move freely. As a result, the impedance or resistance of the nanocomposite increased. With further increasing of RH, occurrence of the physical adsorption (physisorption) of water molecules onto the available active sites of the surface of oxide based CaMgFe_1.33_Ti_3_O_12_ nanoceramic derived sintered electro-ceramic nanocomposite was found via single hydrogen bonding (Figure 13). Due to single hydrogen bonding, the water molecules became mobile and thus conductivity increased progressively; further raising of RH increased the multilayer physical adsorption; and the physisorbed water molecules were ionized by the application of external electric field, and as a result, a large number of hydronium ions (H_3_O^+^) might be available for conduction [65].

**Figure 14:**
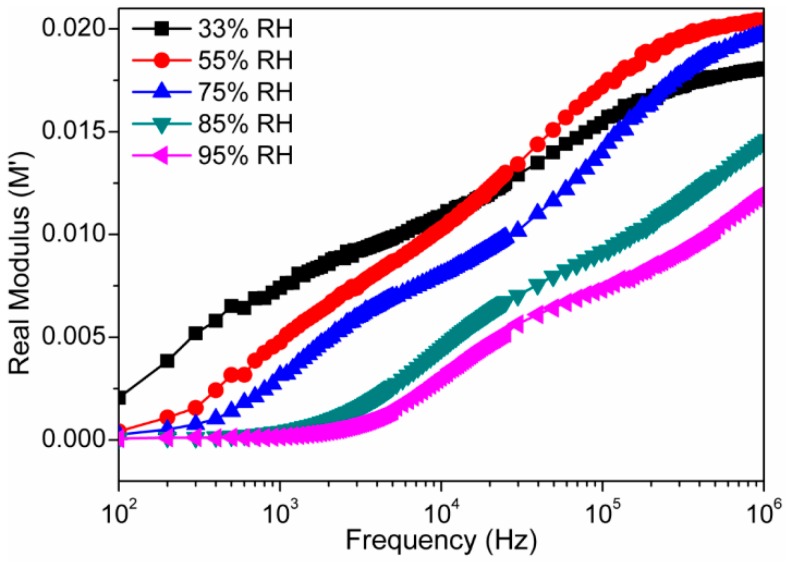
The variation of real (M′) modulus components of the electro-ceramic nanocomposite at different RH as a function of frequency in logarithmic scale at 25 °C. Note: A sigmoidal shape curve is observed, when the value of M′ changes from low to high RH value. This is mainly due to the existence of relaxation phenomena inside the newly developed electro-ceramic nanocomposite sensor at the time of interaction to the water particles.

**Figure 15:**
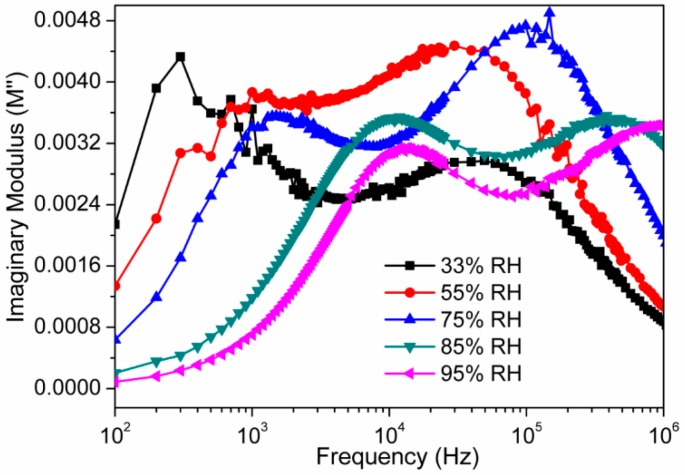
Variations in imaginary (M") modulus components of the sensor based on electro-ceramic nanocomposite at different RH as a function of frequency in logarithmic scale at 25 °C. Note: The relaxation peak frequencies are shifted in the direction of higher frequencies as the relative humidity increased. The shifting of peaks towards higher frequencies is a clear indication of an increase in direct current (DC) conductivity with relative humidity.

The complex electric modulus (M*) can be expressed using Equation (5) [66]:

(5) $$M^{*}\left(\omega \right)=\frac{1}{{є}^{*}\left(\omega \right)}=M\prime +jM\prime \prime ={j\omega C}_{0}Z^{*}$$

where M′ is a real part of modulus (M′ = jωC_0_Z′), M″ is an imaginary part of modulus (M″ = jωC_0_Z″), ε* is permittivity, Z* is complex impedance, ω is the angular frequency, i.e., ω *= 2*π*f*, *f* is linear frequency, and Z′ and Z″ are the real part and imaginary parts of impedance, respectively. C_0_ is the geometrical capacitance, i.e., C_0_ = ϵ_o_A/*t*, where A is area of the electrode, *t* is thickness and ϵ_o_ is permittivity of air. To suppress the electrode polarization and space charge injection phenomena, the complex modulus analysis is an effective method. The frequency dependent M′ and M″ responses at different RH are shown in Figure 14 and Figure 15, respectively. In Figure 14, when the value of M′ changed from low to high, a sigmoidal shape curve was observed. This observation confirms the existence of relaxation phenomena inside the newly developed nanocomposite at the time of interaction to the water particles. This observed phenomena can be accompanied by a loss peak in log(*f*) vs. M″ plot, as shown in Figure 15. The existence of relaxation peaks was confirmed by plots depicted in the Figure 15. It had been observed that the relaxation peak frequencies were shifted toward higher frequencies as the relative humidity increased. The shifting of peaks towards higher frequencies suggests the increase in DC conductivity with relative humidity [50]. The frequency where the peak is formed, is known as relaxation frequency and the corresponding time is known as relaxation time, i.e., τ = ½π*f_max_*, where *f_max_* is peak frequency. Since the relaxation frequency is increased with increased RH, the relaxation time decreases with the increase in humidity.

**Figure 16:**
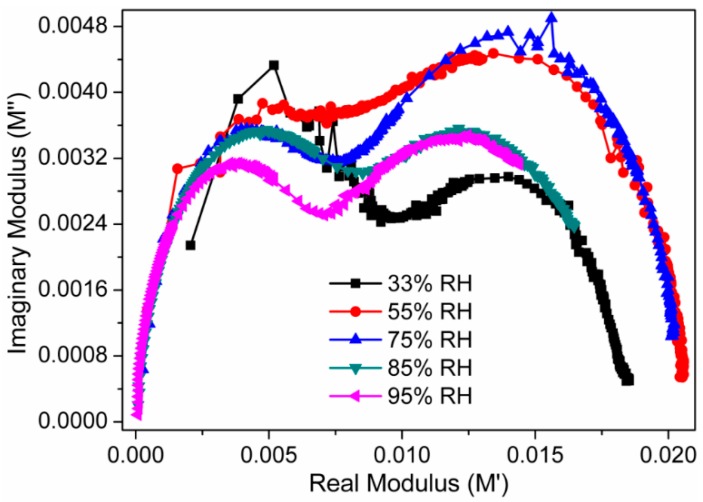
Complex modulus responses between real (M′) and imaginary (M″) at different RH of the sensor based on sintered electro-ceramic nanocomposite at 25 °C. Note: The radius of the semicircle decreases, with increased relative humidity, which suggests that the bulk resistance of the electro-ceramic nanocomposite humidity sensing device decreases with the increase in RH.

The complex modulus spectra (M′ vs. M″) of the present electro-ceramic nanocomposite sensor device at different relative humidity are depicted in Figure 16. The modulus data highlighted in the complex modulus plots help to understand the conductivity relaxation phenomena in terms of the change in M″ with frequency. The asymmetric semicircular arc observed in this complex modulus plot ensures the contribution of relaxation phenomena in the present electro-ceramic nanocomposite sensor system. As the relative humidity increases, radius of the semicircle decreases. It indicates that the bulk resistance of the present CaMgFe_1.33_Ti_3_O_12_ nanoceramic derived sintered electro-ceramic nanocomposites based humidity sensing device decreases with the increase in relative humidity.

## 4. Conclusions

This investigation successfully fabricated impedance or resistive type humidity sensor based on CaMgFe_1.33_Ti_3_O_12_ nanoceramic derived porous sintered electro-ceramic nanocomposites by using solid-state step-sintering. The typical structures and morphology were found by XRD and SEM. The desired grain size, high porosity and good surface-hydrophilicity properties were obtained for the nanocomposites sintered at 1050 °C. The sintered electro-ceramic exhibited excellent humidity sensing properties, including high sensitivity, faster response (20 s) and recovery (40 s) times, narrow hysteresis (<3.4%), and good stability over a long period. These advanced improved characteristics were attributed to suitable stable grain size and porous nature of the nanocomposite. The obtained electrical results confirm that our newly developed CaMgFe_1.33_Ti_3_O_12_ nanoceramic derived sintered electro-ceramic nanocomposite material based humidity sensor is better than many currently existing conventional metal oxide based humidity sensors [51,54,55,56,57,58,59,60,61]. The obtained best performance of the present sintered electro-ceramic nanocomposite based humidity sensor is due to desired grain size, highly hydrophilic and desired porous nature at the sintering condition of 1050 °C. The sensing properties of our present electro-ceramic based impedance type humidity sensor can be further improved by varying composition and sintering conditions according to desired properties for specific application. Therefore, the present novel fabrication technique and the newly developed sintered electro-ceramic nanocomposite would be very promising for advanced humidity sensor applications. Moreover, since this sensor showed excellent sensitivity and could be able to make thinner less than 1 mm [8], this material has potential for use in advanced humidity sensors for biomedical applications [41], particularly at skin–socket interfaces of an artificial stump.

## Figures and Tables

**Table 1:** Concentration of crystalline phases present the sintered nanocomposite determined from XRD.

Crystalline Phase	Area under XRD Peak (s)	Phase Concentration (%)
Fe_2_MgTi_3_O_10_	140,066	85.80
CaTiO_3_	19,857	12.16
Fe_3_O_4_	3319	2.04

**Table 2:** Comparison of lattice parameters of the two phases of sintered nanocomposites with the standard crystals.

Lattice Parameters	Fe_2_MgTi_3_O_10_ (Calculated)	PDF No. 00-013-0353 (Standard)	CaTiO_3_ (Calculated)	PDF No. 00-008-0092 (Standard)
*a* (nm)	0.9658	0.9770	1.5164	1.5250
*b* (nm)	1.0046	0.9950	-	-
*c* (nm)	0.3725	0.3730	-	-
Volume (nm^3^)	0.3614	0.3626	3.4869	3.5466
Lattice volume strain (%)	−0.3309	-	−1.6833	-
Crystal system	Orthorhombic	Orthorhombic	Cubic	Cubic
Crystallite size (nm)	20.7	-	5.9	*-*
